# États de stress en consultation externe à l’hôpital général de Bangui dans un contexte de conflit arme: étude transversale descriptive

**DOI:** 10.11604/pamj.2018.29.26.7107

**Published:** 2018-01-12

**Authors:** Magloire Nkosi Mpembi, Thierry Lukeba, Damien Mayemba, Victoria Kubuta Massamba, Thierry Matonda ma Nzuzi, Symphorien Gokara, Etienne Vermeiren, Gilbert Mananga Lelo, Samuel Mampunza ma Miezi

**Affiliations:** 1 Département de Psychiatrie, Université de Kinshasa, Republique Democratique du Congo; 2 Médecins sans Frontières, Kinshasa, Republique Democratique du Congo; 3 Centre Neuro Psycho Pathologique de l'Université de Kinshasa, Republique Democratique du Congo; 4 Axe Santé des Populations et Pratiques Optimales en Santé, Centre de Recherche du CHU de Québec, Hôpital du Saint-Sacrement, Québec, Canada; 5 Neurophysiology Unit, GIGA Neurosciences, University of Liège, B-4000 Liège, Belgium; 6 Unité de Crise, Services des Urgences Psychiatrique, Cliniques Universitaires Saint Luc, Université Catholique de Louvain, Belgique

**Keywords:** États de stress, état de stress post-traumatique, conflit, soins ambulatoires, République Centrafricaine, Stress syndrome, post-traumatic stress disorder, conflict, outpatient care, Central African Republic

## Abstract

**Introduction:**

dans les zones de conflit en Afrique les récentes études rapportent des fréquences élevées des états de stress posttraumatiques (ESPT) notamment en milieu communautaire. L'objectif général de cette étude est de contribuer à une meilleure prise en charge des patients confrontés à la violence subséquente au conflit sociopolitique en cours en République centrafricaine.

**Méthodes:**

il s'agit d'une étude transversale portant sur les dossiers des patients reçus en consultation externe Trauma center de Médecins sans Frontières/France à Bangui.

**Résultats:**

l'ESPT était présent chez 33.33 % (n = 35) alors que l'état de stress aigu était présent chez 17.14 % (n = 18) des patients. Les états de stress (ES) étaient associés au sexe féminin, au viol, à l'anxiété et à la dépression. Le viol multipliait par 8 le risque de survenue d'un ES. L'âge médian observé était de 30 ans (P25: 22 ans; P75: 40 ans). La majorité des patients présentait un trouble de l'humeur (63.81 %; n = 67). L'insomnie était présente chez 62.83 % (n = 66). L'anxiété (HADS) était présente chez 44.76 % des patients (n = 47). La dépression a été retrouvée chez 40.95 % (n = 43).

**Conclusion:**

les résultats obtenus montrent à quel point, au-delà des membres de milices, la société est touchée par la violence du conflit que traverse le pays. Ces résultats pourraient nourrir la réflexion sur l'organisation des soins et la prise en charge de la population centrafricaine considérant l'impact à court, moyen et long terme des états de stress aigus en situation de conflit.

## Abstract (en)

**Context:**

in Africa's zones of conflict, recent studies report a high frequency of post-traumatic stress disorder (PTSD) particularly in community settings.

**Objective:**

This study aimed to contribute to a better management of patients experiencing violence subsequent to the Central African Republic socio-political conflict.

**Material and methods:**

We conducted a cross-sectional study of the medical records of patients receiving outpatient treatment in the Doctors Without Borders/Médecins Sans Frontières (France) Trauma Center, Bangui.

**Results:**

33.33% (n=35) of patients had PTSD, while 17.14% (n=18) of patients had acute stress syndrome. Stress syndrome (SS) was associated with female sex, rape, anxiety and depression. Rape multiplied the risk of SS occurrence by 8. The average age was 30 years (P25:22 years; P75:40 years). The majority of patients had mood disorder (63.81%; n=67). Insomnia was present in 62.83% (n=66) of patients. Hospital Anxiety and Depression Scale (HADS) was present in 44.76 % of patients. Depression was found in 40.95% (n=43) of patients.

**Conclusion:**

The obtained results show how the society, apart from militia members, is affected by conflict-related violence in the country. These results can enrich the reflections on health organisation and on the management of patients in Central African, by considering the impact of conflict-related acute stress syndome in the short, medium and long term.

## Introduction

Face à un évènement stressant le DSM V [1] répertorie les réponses ci-après : l'état de stress post-traumatique (posttraumatic stress disorder) et l'état de stress aigu (acute stress disorder) qui font l'objet du présent travail, le trouble réactionnel de l'attachement (reactive attachment disorder), l'état de désinhibition dans les contacts sociaux (disinhibited social engagement disorder) ainsi que le trouble de l'adaptation (adjustement disorders). Le diagnostic des états de stress (ES) post-traumatique (ESPT) et aigu (ESA) repose sur un certain nombre de critères. Les symptômes regroupés en syndrome de répétition, syndrome d'évitement persistant et syndrome d'hyperéveil surviennent dans un contexte où l'individu a vécu, a été témoin ou a été confronté à un évènement ou à des évènements durant lesquels des individus ont pu mourir ou être très gravement blessés, ou bien ont été menacés de mort ou de graves blessures, ou bien durant lesquels son intégrité physique ou celle d'autrui a pu être menacée avec comme réaction de l'individu une peur intense, un sentiment d'impuissance ou d'horreur [2]. Cette dernière condition n'est cependant pas requise dans la dernière édition du DSM (1). Les données sur la prévalence des ESPT au sein de la population générale proviennent principalement des études menées aux USA [2]. Elle serait de 1.2 % parmi les hommes et de 2.7 % parmi les femmes le premier mois après le traumatisme [3]. Ce taux monte à 5-6 % chez les hommes et à 10-12 % chez la femme lorsque l'on considère la prévalence sur toute la vie [4]. En Europe, les enquêtes menées montrent une prévalence plus faible autour de 0.9 % pour les hommes et de 2.9 % chez les femmes [2].

En Afrique subsaharienne de récentes enquêtes ont été menées auprès des populations spécifiques ou dans des zones de conflit. Dans une étude ayant pour objectif de déterminer les rapports entre les agressions sexuelles et les troubles de santé mentale à l'est de la République Démocratique du Congo, Johnson et al. ont rapporté une prévalence de 50.1 % [5]. Un taux de 34.9 % d'ESPT a été retrouvé auprès des ex-enfants-soldats en Ouganda et en République démocratique du Congo [6]. Le potentiel invalidant des ESPT est aujourd'hui bien documenté [7]. Les enquêtes menées auprès des vétérans montrent que l'impact des évènements traumatisants demeure présent de nombreuses années après la survenue du traumatisme [8]. En République Centrafricaine, il n'existe pas à notre connaissance, d'études évaluant l'ampleur des conséquences psychopathologiques du conflit sociopolitique en cours notamment en ce qui concerne les ESPT. Ce pays ne dispose pas non plus d'un personnel qualifié en nombre suffisant pour prendre en charge les patients en situation de détresse psychologique. L'objectif général poursuivi dans cette étude est de contribuer à une meilleure prise en charge des patients confrontés à la violence subséquente au conflit sociopolitique en cours en République centrafricaine. Les objectifs spécifiques assignés sont d'évaluer la prévalence des ES et d'en déterminer les facteurs sociodémographiques et cliniques associés.

## Méthodes

**Type d'étude et population:** Il s'agit d'une étude transversale portant sur les dossiers des patients reçus en consultation externe (n=105) entre le 15 juillet et le 10 octobre 2014 à au Trauma center de Médecins sans Frontières/France à l'hôpital général de Bangui. Les dossiers incomplets et ceux des patients âgés de moins de 18 ans n'ont pas été considérés pour la présente étude.

**Outils d'évaluation clinique:** Le dossier médical comprenait l'anamnèse et l'observation psychiatrique. Chaque patient a répondu au questionnaire de l'échelle de changement de vie de Holmes et Rahe [9]. L'Hospital Anxiety Depression Scale (HADS) de Sigmond et Snaith [10] a servi évaluer la sévérité de l'anxiété et de la dépression. Les états de stress (ES) ont été dépistés et évalués avec l'Impact of Event scale revised (IES) de Weiss et Marmarr [11].

**Analyses statistiques:** Les données ont été analysées avec le logiciel Epi info 6.04 version française. Dans un premier temps, les résultats des analyses descriptives ont été présentés sous forme de fréquences pour les variables qualitatives et sous forme de valeur médiane avec P25 et P75 pour les variables quantitatives non normalement distribués. La moyenne ± écart-type a été utilisée à des fins de comparaison. Pour faciliter l'analyse, les résultats ont été dichotomisés (Tableau 1). Dans un deuxième temps, en analyse bivariée, les recherches d'association entre différentes variables ont été réalisées en utilisant les tables de comparaisons des proportions et le test de Chi2 de Pearson. Le seuil de signification statistique retenu était de 5 %. Dans un troisième temps, afin de déterminer les facteurs de risque des ES, un modèle a été construit à l'aide de la régression logistique. Ont été introduits dans le modèle les facteurs qui en analyse univariée se sont avérés associés de manière significative aux états de stress.

**Tableau 1:** Catégories dichotomiques utilisées pour l’analyse des résultats

Variables	Catégories	Critères	Effectif (N)	Pourcentage (%)
Sexe	Hommes	Sexe mâle	59	56,19
	Femmes	Sexe femelle	46	43,81
Enfants à charge	Absents	0 enfant à charge	36	34,29
	Présents	1 enfant ou plus à charge	69	65,71
État civil	En couple	Vit maritalement	48	45,71
	Célibataire	Vit seule	57	54,29
Âge	Jeune	≤ 29 ans	50	47,62
	Ainés	≥ 30 ans	55	52,38
Alcool	Consommation	Oui	27	25,71
	Pas de consommation	Non	78	74,29
Tabac	Consommation	Oui	86	81,9
	Pas de consommation	Non	19	18,1
Cannabis	Pas de consommation	Oui	100	95,24
	Consommation	Non	5	4,76
Durée Trauma	Trauma récent	< 1 mois	57	54,29
	Trauma aigu	≥ 1mois	48	45,71
Anxiété	Absence anxiété	score ≤ 10	58	55,24
	État anxieux	Score > 10	47	44,76
Dépression	Absence dépression	score ≤ 10	62	59,05
	État dépressif	Score > 10	43	40,95
Niveau de stress	Stress modéré	score < 150	52	49,52
	stress élevé	score ≥ 150	53	50,48

## Résultats

**Fréquence des ES:** A l'IES, la moitié des patients (50.48 %; n = 53) présentait un ES. L'ESPT était présent chez 33.33 % (n = 35) et l'ESA chez 17.14 % (n = 18).

**Caractéristiques sociodémographiques:** Les caractéristiques sociodémographiques sont présentées dans le Tableau 2. Le sex ratio était de 1.28 en faveur du sexe masculin. L'âge médian observé était de 30 ans (P25: 22 ans; P75: 40 ans). La moyenne était de 32.64±13.09 ans. Près de 3 sujets sur 5 (58.1 %) étaient âgés de 32 ans ou moins.

**Tableau 2:** Caractéristiques sociodémographiques des sujets de l’étude

Paramètres	Effectif (n)	Pourcentage (%)
**Sexe**		
Homme	59	56,19
Femme	46	43,81
**Âge (années)**		
18-24	37	29,52
25-34	29	27,62
35-44	22	20,95
45-54	9	8,57
55-64	5	4,76
65-74	3	2,86
**Adresses**		
Bangui Chrétien	71	67,62
Bangui Musulmans	19	18,1
Hors Bangui Chrétien	7	6,67
Hors Bangui Musulmans	1	0,95
Hors Bangui Mixte	7	6,67
**État civil**		
En couple	48	45,71
Célibataire	57	54,29
**Enfants en charge**		
0	36	34,29
1-5	57	54,29
6-10	9	8,57
10 et plus	3	2,86

**Habitudes de consommation:** Le Tableau 3 montre les habitudes de consommation. L'alcool, le tabac et le cannabis étaient respectivement consommés par 25.71 % (n = 27), 18.1 % (n = 19) et 4.76 % (n = 5).

**Tableau 3:** Habitudes de consommation

Substances	Effectif (N)	Pourcentage (%)
**Alcool**		
oui	27	25,71
non	78	74,29
**tabac**		
oui	19	18,1
non	86	81,9
**Cannabis**		
oui	5	4,76
non	100	95,24

**Types de traumatisme:** Le Tableau 4 montre les différents types de traumatismes rapportés par les patients. Les viols et les actes de torture-bastonnade étaient les traumatismes les plus fréquents à hauteur respective de 21.9 % (n=23) et de 27.62 % (n=29).

**Tableau 4:** Types de traumatisme rapporté

Traumatismes	Effectif (N)	Pourcentage (%)
Plaie par balle	11	10,48
Plaie par arme blanche	2	1,9
Torture bastonnade	29	27,62
Viols	23	21,9
Autres	40	38,1

**Caractéristiques cliniques:** Les caractéristiques cliniques des patients sont sur le Tableau 5. La majorité des patients présentait un trouble de l'humeur (63.81 %; n = 67). L'insomnie était présente chez 62.83 % (n = 66). A l'échelle de Holmes et Rahe la moitié des patients (50.48 %; n = 53) affichait un score supérieur ou égale à 150. L'anxiété (HADS) était présente chez 44.76 % des patients (n = 47). La dépression a été retrouvée chez 40.95 % (n = 43).

**Tableau 5:** Caractéristiques cliniques

Signes/symptômes	Effectif (N)	Pourcentage(%)
Cauchemars	38	36,19
Peur (Panique)	42	40
Insomnie	66	62,86
Trouble de l’humeur	67	63,81
Trouble dans le contact	14	13,33
Trouble appétit	35	33,33
**Niveau de stress (Échelle de Holmes et Rahe)**		
score < 150	52	49,52
score ≥ 150	53	50,48
**Anxiété (Hospital anxiety depressionscale Scale)**		
score ≤ 10	58	55,24
score > 10	47	44,76
**Depression (Hospital anxiety depressionscale Scale)**		
score ≤ 10	62	59,05
score > 10	43	40,95
**Etats de stress (Impact of Event Scale Revised de Weiss et Marmar)**		
Score < 33	52	49,52
Score ≥ 33		
ESPT	35	33,33
ESA	18	17,14
**Total États de stress**	53	50,48

**Facteurs associés aux ES:** Les ES étaient associés entre autres au sexe féminin, au viol, à l'anxiété et à la dépression (Tableau 6). En même temps, le viol, l'insomnie, les cauchemars et le niveau de stress élevé multipliaient le risque de survenue des ES respectivement par 7.97, 9.37, 7.74 et 3.81 (Tableau 7).

**Tableau 6:** Relations entre les facteurs sociodémographiques, les habitudes de consommation, le type de traumatisme, les caractéristiques cliniques et les états de stress

Facteurs	p
**Facteurs sociodémographiques**	
Sexe féminin	
ESA	NS
ESPT	0,035
Âge	NS
État civil	NS
Enfants à charge	NS
**Habitudes de consommation**	
Alcool	NS
Tabac	NS
Chanvre	NS
**Types de traumatisme**	
Viol	< 0,01
ESA	< 0,01
ESPT	NS
Torture	0,02
ESA	< 0,01
ESPT	NS
Plaie par balle	NS
Plaie par arme blanche	NS
**Caractéristiques cliniques**	
Cauchemars	< 0,01
ESA	< 0,01
ESPT	< 0,01
Peur/Panique	< 0,01
ESA	< 0,01
ESPT	< 0,01
**Trouble du sommeil**	0,00
ESA	< 0,01
ESPT	< 0,01
**Troubles de l’appétit**	< 0,01
ESA	< 0,01
ESPT	< 0,01
**Niveau de stress élevé**	< 0,01
ESA	0,02
ESPT	NS
Anxiété	0,00
ESA	< 0,01
ESPT	< 0,01
Dépression	0,00
ESA	< 0,01
ESPT	< 0,01

**Tableau 7:** Facteurs de risque de survenue des États de stress

Variables	Références	OR ajustés	IC 95 %	P
Viol	Pas de viol	7,97	1,34-47,53	0,02
Insomnie	Absence d’insomnie	9,37	2,34-37,59	< 0,01
Cauchemars	Absences de cauchemars	7,74	1,91-31,39	< 0,01
Niveau de stress	Niveau de stress <150	3,81	1,18-12,31	0,03

## Discussion

La présente étude avait pour objectif d'évaluer la prévalence des ES et d'en déterminer les facteurs sociodémographiques et cliniques associés en consultation externe à l'Hôpital général de Bangui. La fréquence observée dans le cadre de cette étude était de 50.48 % dont 33.33 % pour les ESPT et 17.14 % pour les ESA. Ce résultat est comparable à ceux rapportés par les études menées auprès des sujets vivant en zones de conflit en Afrique aussi bien en communauté [5, 12,13] qu'en milieu hospitalier [14,15]. Il est le témoin de l'ampleur de la violence vécue par les populations en République centrafricaine. On a noté une surreprésentation du sexe masculin. Elle serait liée au fait que les hommes sont plus directement impliqués dans le conflit en tant qu'acteurs ou victimes, les milices actives étant en général majoritairement composées des hommes. Une grande proportion des sujets étaient âgés de moins de 32 ans. Ce résultat traduit le rôle joué par les jeunes dans le conflit. Il est aussi le reflet de la jeunesse de la population centrafricaine [16]. La diffusion de la violence au sein de la société civile est mise en lumière par le fait que les viols, les actes de torture et de bastonnade comptaient parmi les traumatismes les plus retrouvés. En effet bon nombre des personnes victimes n'étaient pas affiliées à des milices combattantes. Par ailleurs, il est intéressant de noter que le rôle de la torture dans la survenue des ESPT en situation de post-conflit a été mis en lumière dans une étude multicentrique menée en Algérie, au Cambodge, en Éthiopie et à Gaza [17]. La fréquence élevée des troubles de l'humeur retrouvée dans l'étude notamment la dépression correspond aux données de la littérature. En Ouganda Vinck et al. ont observé une prévalence de 44.5 % dans une étude menée dans les villages et camps des refugiés auprès des populations exposées à des crimes de guerre [18]. Dans la même région, Roberts et al. ont rapporté une prévalence de 54 % pour l'ESPT et de 67 % pour la dépression [19]. Dans la présente étude les ES étaient associés à au sexe féminin, au viol, à l'anxiété et à la dépression. Les travaux de Roberts et al. ont rapporté des observations similaires en ce qui concerne le rôle du viol ou des abus sexuels dans la survenue des ESPT [19] en Ouganda. Le sexe féminin était particulièrement exposé aux ES en contexte de conflit. Pour les sujets de la présente étude, le viol multipliait par 7.97 le risque de présenter un ES.

## Conclusion

La fréquence des ES parmi les sujets de l'étude était élevée. Le genre et les viols comptaient parmi les facteurs importants associés à la survenue des ES. Les résultats obtenus montrent à quel point la violence touche les différentes couches de la société au-delà des membres de milices. Ces résultats pourraient nourrir la réflexion des autorités sanitaires centrafricaines sur l'organisation des soins et la prise en charge de la population centrafricaine en santé mentale considérant l'impact à court, moyen et long terme des états de stress aigus en situation de conflit.

### Etat des connaissances actuelles sur le sujet

- La prévalence des états de stress post-traumatiques chez les civils ayant été confrontés à une guerre civile en Afrique est très élevée selon la littérature disponible;
- Les études épidémiologiques sur les états de stress post-traumatiques sont généralement menées à distance du conflit.

#### Contribution de notre étude à la connaissance

- Cette étude évalue l'impact psychopathologique du conflit centrafricain sur les populations non combattantes dans l'immédiat post-conflit;
- Cette étude met en lumière le tribut psychopathologique important payé par les femmes centrafricaines dans le conflit;
- Les résultats obtenus peuvent servir de support pour l'élaboration des programmes de prise en charge des problèmes de santé mentale en République centrafricaine.

## Conflits d’intérêts

Les auteurs ne déclarent aucun conflit d'intérêts.

## Remerciements

Les auteurs remercient mesdames Frédérique Drogoul et Hélène Thomas pour leur soutien à la réalisation de cette étude.
